# Does adherence to exacerbation action plans matter? Insights from two COPD self-management studies

**DOI:** 10.1016/j.heliyon.2024.e39070

**Published:** 2024-10-10

**Authors:** Jade Schrijver, Tanja Effing, Joanke van Helden, Job van der Palen, Paul van der Valk, Marjolein Brusse-Keizer, Anke Lenferink

**Affiliations:** a Cognition, Data and Education, Faculty of Behavioural, Management and Social Sciences, University of Twente, Enschede, the Netherlands; b Department of Pulmonary Medicine, Medisch Spectrum Twente, Enschede, the Netherlands; c College of Medicine and Public Health, Flinders University, Adelaide, Australia; d School of Psychology, Faculty of Health and Medical Sciences, University of Adelaide, Adelaide, Australia; e Health Technology and Services Research, Faculty of Behavioural, Management and Social Sciences, Technical Medical Centre, University of Twente, Enschede, the Netherlands; f Medical School Twente, Medisch Spectrum Twente, Enschede, the Netherlands; g Clinical Research Centre, Rijnstate Hospital, Arnhem, the Netherlands

**Keywords:** Chronic obstructive pulmonary disease, Adherence, Action planning, Self-treatment, Exacerbations, Health outcomes

## Abstract

**Introduction:**

Patients' adherence is essential for COPD self-management, as beneficial effects can only be expected in adherent patients. We explored associations between patients’ adherence to COPD exacerbation action plans and health outcomes.

**Materials and methods:**

Pooled COPD self-treatment intervention group data from two RCTs were analysed, only including patients who had ≥1 COPD exacerbation or started ≥1 course of oral corticosteroids over one-year follow-up. Optimal adherence was defined as ‘self-treatment initiated ≤1 day before or after exacerbation start’, suboptimal adherence as ‘self-treatment initiated 2 days before or after exacerbation start or no self-treatment initiated for a short (1–3 days) exacerbation’, and significant delay or no treatment as ‘self-treatment initiated >2 days after exacerbation start or no self-treatment initiated for a longer (>3 days) exacerbation’. Regression models were built for several health outcomes, with the number of COPD exacerbation days/patient/year being the primary outcome.

**Results:**

Patients with significant delay or no treatment (n = 46) had more exacerbation days/patient/year (33.3 (95 % CI 10.9; 55.6)) than optimal adherent patients (n = 38) (23.7 (95 % CI 1.7; 45.7)). The duration per COPD exacerbation was longer for patients with significant delay or no treatment (15.5 days) compared to optimal adherent patients (7.8 days). No differences in health outcomes were observed between optimal and suboptimal adherent patients.

**Conclusions:**

Being adherent to action plans is associated with better health outcomes than significant delayed treatment or no treatment at all. Interestingly, suboptimal adherence demonstrated health benefits comparable to optimal adherence. COPD self-management interventions should prioritise strategies to optimise patients’ adherence to action plans.

## Highlights

• Being (sub)optimal adherent to action plans is associated with health benefits.
• Delayed or no treatment is associated with having more COPD exacerbation days.
• COPD self-management interventions should include strategies to optimise adherence.

## 1 Introduction

Chronic Obstructive Pulmonary Disease (COPD) is a progressive lung condition [1]. COPD patients typically experience exacerbations that, apart from a social and economic burden, have a large impact on the patient's well-being [2,3]. Self-management interventions are pivotal in COPD patients' disease management [4,5], as they lead to improved health outcomes, including improved quality of life, reduced respiratory-related hospital admissions and healthcare utilisation [6]. Action planning is an important behavioural change technique in COPD self-management interventions [7,8]. A COPD exacerbation action plan encourages patients to focus on early recognition of respiratory symptoms and to start prompt actions when a COPD exacerbation develops (e.g. starting self-treatment with corticosteroids, contacting a healthcare professional for support) [9,10]. Using action plans in COPD self-management interventions potentially reduces exacerbation duration, hospitalisation rate, and healthcare costs, and it may improve COPD patients' quality of life [[10], [11], [12]].

Patients' adherence is crucial for interventions to be effective [13]. Self-management interventions are indeed more likely to lead to improved health outcomes (e.g. reduction in exacerbation duration and hospitalisations) if patients adhere to their exacerbation action plans [[14], [15], [16]]. However, only 40 % of COPD patients seem to be adherent to action plans [15,17,18], and little is known regarding associations between non-optimal adherence and health outcomes. Previous studies used various definitions of adherence, e.g. based on 1) time difference between exacerbation start and initiation of a corticosteroids and/or antibiotics course [15,16]; and 2) an evaluation of patients' self-management actions (e.g. using medication, avoiding triggers, contacting healthcare professionals) [14]. In a previously published study, we explored patients' adherence to action plans by incorporating four adherence categories based on the timing of patients' self-treatment actions [18]. Results from this study prompted questions regarding the need for full patient adherence to the action plan. Should patients adhere strictly in terms of timing of self-treatment according to their action plan to derive benefit from it, or can we allow some flexibility in its use (e.g. suboptimal adherence) while still achieving positive health outcomes? The aim of this study was to explore associations between patients' adherence categories and health outcomes. Study findings can provide direction toward tailoring of self-management exacerbation action plans to patients’ needs, preferences, and competencies.

## 2 Material and methods

### 2.1 Study design and population

We conducted secondary analyses to explore associations between patients’ adherence categories and health outcomes, using pooled quantitative data from two randomised controlled trials (RCTs): the COPE-II (2004–2006) [12] and COPE-III study (2012–2016) [11,19]. Both studies evaluated the effectiveness of a self-management intervention including COPD exacerbation action plans [11,12].

The two COPE studies [11,12,19] had partly similar in- and exclusion criteria: a diagnosis of COPD according to the GOLD criteria [20], ≥40 years old, ≥3 COPD exacerbations or ≥1 COPD hospitalisation in the last two years preceding study entry, and having stable disease at inclusion. Whereas patients with severe comorbidities were excluded from the COPE-II study [12], COPE-III study patients had to have at least one comorbidity (ischemic heart disease, chronic heart failure, diabetes mellitus, anxiety, and depression) [11,19].

In this analysis, we only included those patients of the COPE-II [12] and COPE-III study [11,19] who 1) were assigned to the self-treatment intervention groups as we required data from patients who had the opportunity to actively use the COPD exacerbation action plans; and 2) experienced at least one COPD exacerbation or had at least one self-treatment action (starting a self-initiated course of oral corticosteroids) reported during the first year of follow-up.

### 2.2 Self-treatment intervention

The self-treatment interventions in both COPE studies included self-management training sessions directed toward symptom recognition and monitoring, and self-treatment of COPD exacerbations [11,12,19]. Patients received a ‘What are my usual symptoms’ card, that detailed their personal respiratory symptom levels in a stable health state [11,12,19]. Furthermore, they received a daily symptom diary that was linked to a COPD exacerbation action plan [11,12,19]. In case symptoms worsened, patients were asked to follow the instructions defined in the action plan, such as taking medication, or seeking help from a healthcare professional [11,12,19]. When a patient experienced a significant negative change of at least two major symptoms (sputum production, sputum colour, breathlessness) or one major and one minor respiratory symptom (fever, coughing, wheezing) for two consecutive days [21,22], patients were instructed to start a course of oral corticosteroids (and if indicated, an antibiotic course at the same time) [11,12,19]. Patients were also asked to contact their case-manager by phone if they did not feel better two days after initiation of self-treatment or if they had any questions or doubts [11].

### 2.3 Measures

#### 2.3.1 Patients’ adherence

Data on patients' adherence to COPD exacerbation action plans were extracted from patients' symptom diaries (i.e. start and end (dates) of a COPD exacerbation and self-reported initiated courses of oral corticosteroids for the self-treatment of a COPD exacerbation). Adherence was assessed by defining the time between the start of the exacerbation and the initiated self-treatment action (Table 1). The start of a COPD exacerbation was defined as “a significant negative change in two major symptoms or one major and one minor symptom from the usual symptom state for at least two consecutive days” [21,22]. The COPD exacerbation end was defined as the first day of: 1) three successive days that the patient had returned to his usual symptom state; or 2) seven consecutive days on which the patient continuously reported no or only a slight increase in symptoms compared to baseline, with no fever or change in sputum colour [11,12,19]. The following four adherence categories were defined a-priori: 1) optimal adherence: self-treatment initiated ≤1 day before or after exacerbation start; 2) suboptimal adherence: self-treatment initiated 2 days before or after exacerbation start or no self-treatment initiated for a short (1–3 days) exacerbation; 3) significant delay or no treatment: self-treatment initiated >2 days after exacerbation start or no self-treatment initiated for a longer (>3 days) exacerbation; and 4) treatment outside the actual exacerbation period: self-treatment initiated ≥3 days before exacerbation start or after the end of the exacerbation (Table 1). Patients’ most frequent adherence behaviour over the one-year follow-up determined their final adherence category classification.

**Table 1:** Classification of patients’ adherence to COPD exacerbation action plans based on the time difference between the self-initiation of a corticosteroid course and the start of a COPD exacerbation

Optimal adherence	Self-treatment initiated ≤1 day before or after COPD exacerbation start
Suboptimal adherence	Self-treatment initiated 2 days before or after COPD exacerbation start
	No self-treatment initiated for a short (1–3 days) COPD exacerbation
Significant delay or no treatment	Self-treatment initiated >2 days after COPD exacerbation start
	No self-treatment initiated for a longer (>3 days) COPD exacerbation
Treatment outside the actual exacerbation period	Self-treatment initiated without the presence of a COPD exacerbation
	Self-treatment initiated ≥3 days before COPD exacerbation start
	Self-treatment initiated after COPD exacerbation end

#### 2.3.2 Health outcome measures

The primary health outcome measure was the number of COPD exacerbation days/patient/year. Secondary outcomes are summarised in Table 2.

**Table 2:** Description of secondary health outcome measures

Outcome measure	Description
Number of COPD exacerbations/patient/year	The number of exacerbations per patient. Retrospectively extracted from the daily symptom diary.
Duration per COPD exacerbation/patient/year	The number of days between the start and end of an exacerbation. Retrospectively extracted from the daily symptom diary.
Health related quality of life	Change from baseline at one year in Chronic Respiratory Questionnaire (CRQ) score [43].
Anxiety and depression symptoms	Change from baseline at one year in Hospital Anxiety and Depression Scale (HADS) score [44,45].
Healthcare utilisation	Number of hospitalisations; number of in-hospital days; number of patients with at least one hospitalisation. Retrospectively extracted from the patients' medical records.
Mortality	Number of all-cause deaths. Retrospectively extracted from the patients' medical records.

### 2.4 Analyses

Statistical analyses were performed using IBM SPSS 26. Descriptive statistics were used to summarise the baseline characteristics and health outcomes as means (SD), medians (interquartile ranges (IQR)) or frequencies (proportions), as appropriate. Univariate crude associations (p<0.10) between adherence categories and health outcomes were explored using Chi-square or Fishers’ exact tests for categorical variables, and ANOVA or Kruskal Wallis tests for continuous variables. When appropriate, post-hoc analyses were performed using the Tukey SD or Holm-Bonferroni correction. Separate multiple linear regression models were built for the continuous health outcomes that were univariably associated with adherence categories, while adjusting for confounders that showed univariate crude associations (p<0.10) with both the adherence categories and health outcomes. Each model was reduced using backward elimination, in which covariates were removed from the regression model sequentially based on the highest p-value until reaching statistical significance (p<0.05) or until no more covariates could be removed. In addition, after removal of each covariate in the regression models, it was provided that the association between adherence categories and the health outcome in question did not substantially change (<10 %). Because the duration per COPD exacerbation was highly skewed, the data was log-transformed prior to the regression analyses, and back-transformed to determine an estimated geometric mean.

We decided a-priori to not include ‘treatment outside the actual exacerbation period’ in regression analyses with an exacerbation-related health outcome as the dependent variable (i.e. number of COPD exacerbation days, number of COPD exacerbations, and duration per COPD exacerbation). The adherence category ‘treatment outside the actual exacerbation period’ was defined as ’patients initiated self-treatment three or more days before exacerbation start, after the end of an exacerbation, or without the presence of an exacerbation’ (Table 1). Exacerbation frequencies of patients assigned to this adherence category were expected to be low and associations with exacerbation-related health outcomes were therefore not anticipated. Inclusion of this category in the regression analyses was therefore not perceived as meaningful.

## 3 Results

**Fig. 1:**
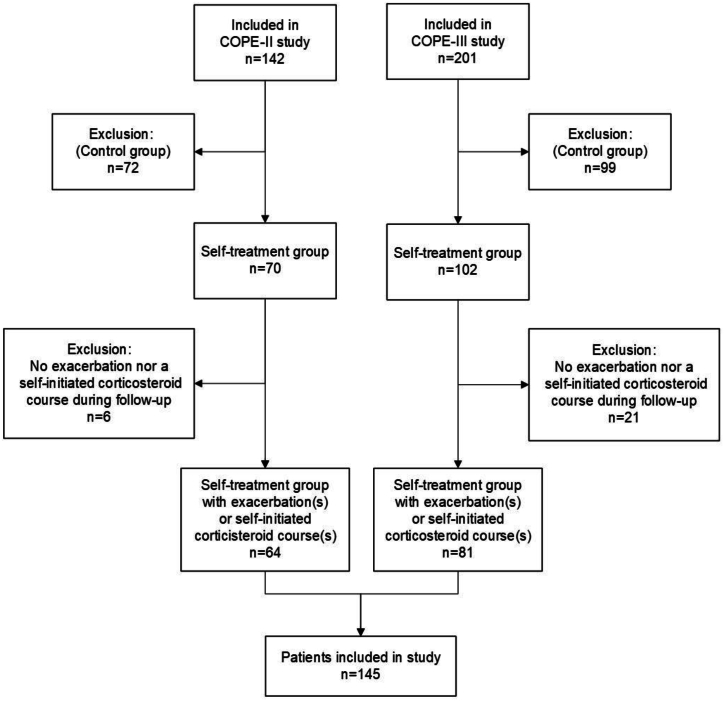
Study flow diagram of the inclusion of COPE-II [12] and COPE-III [11] patient data in secondary analyses.

In total, 145 patients were included in the analyses (COPE-II (n = 64); COPE-III (n = 81)), see Fig. 1. Patients had a mean age of 66.3 (SD 8.8) years, 26.9 % were current smokers, 50.3 % had a low educational level, and 42.8 % had a cardiac comorbidity. For more patient characteristics see Table 3. Thirty-eight (26.2 %) patients were classified as ‘optimal adherent’, 17 (11.7 %) as ‘suboptimal adherent’, 46 (31.7 %) as ‘significant delay or no treatment’, and 44 (30.3 %) as ‘treatment outside the actual exacerbation period’. Patients experienced 438 COPD exacerbations (median per patient 2.0 (IQR 1.0–4.0) (Table 4)) and 127 (87.6 %) patients had at least one exacerbation during the one-year follow-up. A median number of 23 (IQR 8.0–46.5) COPD exacerbation days/patient/year and a median duration per COPD exacerbation/patient/year of 8.7 days (IQR 5.5–14.4) was observed (Table 4).

**Table 3:** Baseline characteristics of patients according to categories of patients’ adherence to COPD exacerbation action plans

	Total (n = 145)	Optimal adherence (n = 38)	Suboptimal adherence (n = 17)	Significant delay or no treatment (n = 46)	Treatment outside the actual exacerbation period (n = 44)	p-value	
						Four adherence categories#	Three adherence categories$
Age (years)	66.3 (8.8)	65.1 (9.6)	66.5 (8.2)	65.5 (9.8)	68.1 (7.1)	0.407	0.881
Men	88 (60.7)	23 (60.5)	11 (64.7)	22 (47.8)	32 (72.7)	0.113	0.356
Body Mass Index	27.4 (24.0–27.0)	25.9 (23.5–30.8)	29.3 (26.6–32.5)	27.5 (23.7–31.8)	27.8 (23.6–31.9)	0.347	0.199
FEV_1_ (l)	1.4 (0.5)	1.5 (0.5)	1.4 (0.5)	1.4 (0.6)	1.3 (0.5)	0.606	0.595
FEV_1_/FVC	46.5 (13.5)	48.1 (15.4)	47.5 (13.3)	47.9 (13.0)	43.3 (12.1)	0.302	0.990
FEV_1_ % predicted	51.1 (16.0)	51.9 (16.6)	51.7 (14.9)	52.4 (17.2)	48.8 (15.0)	0.729	0.988
COPD classification							
GOLD II	75 (51.7)	22 (57.9)	9 (52.9)	25 (54.3)	19 (43.2)	0.595	0.898
GOLD III	56 (38.6)	11 (28.9)	7 (41.2)	16 (34.8)	22 (50.0)		
GOLD IV	14 (9.7)	5 (13.2)	1 (5.9)	5 (10.9)	3 (6.8)		
Current smoker	39 (26.9)	11 (28.9)	3 (17.6)	15 (32.6)	10 (22.7)	0.578	0.507
Educational level^a^							
Low	73 (50.3)	19 (50.0)	8 (47.1)	24 (52.2)	22 (50.0)	0.997	0.964
Middle	58 (40.0)	16 (42.1)	7 (41.2)	17 (37.0)	18 (40.9)		
High	14 (9.7)	3 (7.9)	2 (11.8)	5 (10.9)	4 (9.1)		
CRQ domains							
Dyspnoea	4.3 (1.4)	4.4 (1.6)	4.8 (1.2)	4.2 (1.2)	4.3 (1.4)	0.507	0.317
Fatigue	3.9 (1.2)	4.0 (1.3)	4.4 (0.9)	3.8 (1.1)	3.9 (1.2)	0.369	0.196
Emotional	4.7 (1.2)	4.9 (1.0)	4.8 (1.1)	4.4 (1.2)	4.9 (1.2)	0.199	0.156
Mastery	5.0 (1.1)	5.2 (1.1)	5.2 (1.1)	4.8 (1.1)	5.1 (1.0)	0.206	0.124
HADS domains							
Anxiety	5.0 (3.0–9.5)	4 (2.0–9.0)	5.0 (2.5–6.5)	7.0 (4.0–10.0)	5.0 (3.0–10.0)	0.280	0.136
Depression	5.0 (3.0–9.0)	5.0 (2.0–7.3)	5.0 (2.0–8.0)	6.0 (3.8–11.0)	5.0 (3.0–8.8)	0.145	0.074∗
Dyspnoea score (mMRC)^b^	1.9 (1.1)	1.7 (1.1)	1.7 (1.2)	2.2 (1.1)	1.8 (0.9)	0.083∗	0.057∗
Exacerbations two years prior to study entry^c^	3.0 (2.0–4.8)	3.5 (3.0–5.0)	4.0 (2.0–5.0)	3.0 (2.0–4.0)	3.0 (2.0–4.0)	0.468	0.451
Hospitalisations one year prior to study entry	0.0 (0.0–1.0)	1.0 (0.0–1.0)	0.0 (0.0–1.0)	0.0 (0.0–1.0)	0.0 (0.0–1.0)	0.474	0.294
Cardiac comorbidity	62 (42.8)	15 (39.5)	7 (41.2)	13 (28.3)	27 (61.4)	0.016∗	0.463
Comorbid anxiety^d^	27 (18.6)	7 (18.4)	1 (5.9)	10 21.7)	9 (20.5)	0.529	0.342
Comorbid depression^d^	23 (15.9)	5 (13.2)	0 (0.0)	13 (28.3)	5 (11.4)	0.025∗	0.022∗
Living together	94 (64.8)	23 (60.5)	12 (70.6)	30 (65.2)	29 (65.9)	0.887	0.761
Participation in the COPE-III study	81 (55.9)	20 (52.6)	5 (29.4)	24 (52.2)	32 (72.7)	0.016∗	0.224

Data are presented as mean (standard deviation (SD)), number (%), or median (interquartile range (IQR)). Abbreviations: COPD= Chronic Obstructive Pulmonary Disease; CRQ= Chronic Respiratory Disease Questionnaire [43]; FEV1= Forced Expiratory Volume in 1 s; FVC= Forced (expiratory) Vital Capacity; GOLD = Global Initiative for Chronic Obstructive Lung Disease; HADS= Hospital Anxiety and Depression Scale [44,45]; mMRC = modified Medical Research Council [46].

# Four adherence categories included: optimal adherence, suboptimal adherence, significant delay or no treatment, and treatment outside the actual exacerbation period.

$ Three adherence categories included: optimal adherence, suboptimal adherence, and significant delay or no treatment.

∗ Significantly different (p<0.10) between adherence categories.

a Educational level was classified as low: no school or primary school; middle: secondary school or vocational college; or high: undergraduate or postgraduate.

b MRC dyspnoea scores (range 1–5) as used in COPE-II were converted to mMRC scores (range 0–4).

c Data were missing for the number of exacerbations two years prior to study entry (n = 13).

d Patients were diagnosed with anxiety and/or depression based on HADS scores (≥11) [44,45].

**Table 4:** Health outcomes of patients according to categories of patients’ adherence to COPD exacerbation action plans

	Total (n = 145)	Optimal adherence (n = 38)	Suboptimal adherence (n = 17)	Significant delay or no treatment (n = 46)	Treatment outside the actual exacerbation period (n = 44)	p-value	
						Four adherence categories#	Three adherence categories$
Number of COPD exacerbation days/patient/year (median (IQR))	23 (8.0–46.5)	26.0 (11.8–45.3)	32.0 (12.5–46.0)	52.5 (20.3–94.8)	3.0 (0.0–13.5)	NA	0.012∗
Number of COPD exacerbations/patient/year (median (IQR))	2.0 (1.0–4.0)	3.0 (2.0–5.0)	3.0 (2.0–5.0)	3.0 (1.8–6.0)	1.0 (0.0–2.0)	NA	0.990
Duration per COPD exacerbation/patient/year (median (IQR))	8.7 (5.5–14.4)	7.5 (5.9–11.0)	8.0 (5.9–10.8)	12.7 (8.6–24.2)	4.5 (3.0–7.3)	NA	<0.001∗
CRQ domains (mean change from baseline at one year (n))^a^, ^b^							
Dyspnoea	−0.13 (122)	−0.24 (32)	0.16 (16)	−0.55 (37)	0.25 (37)	0.005∗	NA
Fatigue	0.00 (122)	0.05 (32)	−0.3 (16)	−0.11 (37)	0.09 (37)	0.857	NA
Emotional	0.10 (122)	−0.11 (32)	0.16 (16)	0.31 (37)	0.05 (37)	0.355	NA
Mastery	0.24 (122)	0.00 (32)	0.30 (16)	0.43 (37)	0.24 (37)	0.247	NA
HADS domains (mean change from baseline at one year (n))^a^, ^b^							
Anxiety	−0.50 (122)	0.00 (32)	0.00 (16)	−2.00 (37)	0.00 (37)	0.274	NA
Depression	0.00 (122)	0.00 (32)	0.00 (16)	−1.00 (37)	0.00 (37)	0.421	NA
Number of hospitalisations (mean (SD))	0.6 (1.2)	0.7 (1.5)	0.2 (0.6)	0.8 (1.4)	0.5 (0.8)	0.308	NA
Number of in-hospitalisation days (mean (SD))	5.5 (12.1)	6.7 (14.6)	1.5 (3.7)	8.4 (15.1)	3.0 (6.3)	0.236	NA
Number of patients with at least one hospitalisation (n (%))	44 (30.3)	10 (26.3)	3 (17.6)	18 (39.1)	13 (29.5)	0.355	NA
All-cause mortality (n (%))	4 (2.8)	1 (2.6)	1 (5.9)	2 (4.3)	0 (0.0)	0.472	NA

Abbreviations: COPD= Chronic Obstructive Pulmonary Disease; CRQ= Chronic Respiratory Disease Questionnaire [43]; HADS= Hospital Anxiety and Depression Scale [44,45]; IQR = interquartile range; NA = not applicable; SD= Standard Deviation.

# Four adherence categories included: optimal adherence, suboptimal adherence, significant delay or no treatment, and treatment outside the actual exacerbation period.

$ Three adherence categories included: optimal adherence, suboptimal adherence, and significant delay or no treatment.

∗ Significantly different (p<0.10) between adherence categories.

a Higher scores indicate improvement in CRQ, and worsening in HADS.

b Data were missing for CRQ scores (n = 16), and HADS scores (n = 16).

Based on crude associations of adherence categories with health outcomes (p<0.10), multiple linear regression models were built for the following health outcomes: number of COPD exacerbation days/patient/year, number of COPD exacerbations/patient/year, duration per COPD exacerbation/patient/year, and the dyspnoea Chronic Respiratory Disease Questionnaire (CRQ) domain score. No crude associations were detected between adherence categories and other health outcomes (Table 4). Dyspnoea (mMRC) score, participation in the COPE-III study, and comorbid depression showed to be potential confounders and were included as covariates in the multiple regression models where appropriate.

Table 5 shows the results of the final multiple linear regression models for health outcomes associated with adherence categories. A significantly higher number of COPD exacerbation days/patient/year was observed for patients with ‘significant delay or no treatment’ (33.3 (95 % CI 10.9; 55.6) days) compared to optimal adherent patients (23.7 (95 % CI 1.7; 45.7) days) when adjusted for baseline dyspnoea (mMRC) score (Table 5). No significant difference in COPD exacerbation days/patient/year was observed between ‘optimal’ and ‘suboptimal adherent’ patients.

**Table 5:** Results of final multiple linear regression models for health outcomes associated with categories of patients’ adherence to COPD exacerbation action plans

	Intercept	Suboptimal adherence	Significant delay or no treatment	Treatment outside the actual exacerbation period∗
	β (95 % CI)	β (95 % CI)	β (95 % CI)	β (95 % CI)
**Number of COPD exacerbation days/patient/year**^a^	23.7 (1.7; 45.7)	0.1 (−28.9; 29.2)	33.3 (10.9; 55.6)∗	NA
**Number of COPD exacerbations/patient/year**^a^	3.2 (2.1; 4.3)	−0.1 (−1.5; 1.3)	−0.2 (−1.3; 0.9)	NA
**Duration per COPD exacerbation/patient/year**^b^	7.8	0.1	7.7∗	NA
**CRQ dyspnoea**^c^, ^d^	0.1 (−0.3; 0.4)	0.3 (−0.3; 0.9)	−0.4 (−0.8; 0.1)	0.6 (0.1; 1.1)∗

Optimal adherence was set as reference. Abbreviations: CI= Confidence Interval; COPD= Chronic Obstructive Pulmonary Disease; CRQ= Chronic Respiratory Disease Questionnaire [43]; mMRC = modified Medical Research Council [46]; NA = not applicable; β = regression coefficient.

∗ Significantly different (p<0.05) from optimal adherence.

a Model adjusted for baseline dyspnoea (mMRC) score.

b Data of duration per COPD exacerbation/patient/year was log-transformed in the multiple linear regression analyses, and expressed as geometric means in this table.

c Model adjusted for COPE-III study participation.

d Higher scores indicate improvement in the dyspnoea CRQ domain.

There was no significant difference detected in the number of COPD exacerbations/patient/year between the three adherence categories.

The duration per COPD exacerbation/patient/year was significantly longer for the patients classified as ‘significant delay or no treatment’ (geometric mean 15.5 days) compared to those classified as ‘optimal adherent’ (geometric mean 7.8 days) (Table 5), again with no significant difference between ‘optimal’ and ‘suboptimal adherent’ patients.

Patients with ‘treatment outside the actual exacerbation period’ had a significantly better dyspnoea CRQ domain score (0.6 (95 % CI 0.2; 1.1)) than ‘optimal adherent’ patients (0.1 (95 % CI -0.3; 0.4)) when adjusted for COPE-III study participation (Table 5), and no significant differences were detected between ‘suboptimal adherent’ or ‘significant delay or no treatment’ and ‘optimal adherent’ patients.

## 4 Discussion

Our analyses demonstrate that patients classified as ‘optimal’ and ‘suboptimal adherent’ had fewer COPD exacerbation days and a shorter duration per COPD exacerbation compared to patients classified as ‘significant delay or no treatment’. Furthermore, a significantly better dyspnoea CRQ domain score was observed for patients with ‘treatment outside the actual exacerbation period’ compared to ‘optimal adherent’ patients.

Patients classified into the ‘significant delay or no treatment’ category had more exacerbation days/patient/year and a longer duration per COPD exacerbation compared to ‘optimal adherent’ and ‘suboptimal adherent’ patients. These findings may be explained by the fact that the latter two groups of patients initiated self-treatment promptly (≤2 days before or after exacerbation start), resulting in effective treatment of the exacerbation with oral corticosteroids [23]. This may have led to accelerated recovery of the exacerbation [24], thus fewer exacerbation days, compared to patients who initiated self-treatment with significant delay (>2 days after exacerbation start) or initiated no self-treatment for longer (>3 days) exacerbations. This is in line with previous studies [15,16] reporting a significant and clinically relevant accelerated exacerbation recovery for adherent patients, compared to non-adherent patients. However, these previous studies defined adherence to the action plan as ‘self-treatment initiated within 72 h of exacerbation start’, whereas in our study we distinguished between patients who followed the action plan more strictly (i.e. ‘optimal adherence’ - self-treatment initiated ≤1 day from the actual exacerbation start) and those who followed it less strictly (i.e. ‘suboptimal adherence’ - self-treatment initiated 2 days from the actual exacerbation start).

Interestingly, we observed no significant or clinically relevant differences between ‘optimal’ and ‘suboptimal adherent’ patients regarding the number of COPD exacerbation days and the duration per COPD exacerbation. These findings are of importance, as it implies similar health benefits for both ‘optimal’ and ‘suboptimal adherent’ patients, a differentiation in adherence previously unexplored. Due to the minimal effect sizes observed, post-hoc power analyses were deemed inconsequential. As patients' symptomatology varies widely due to the heterogeneous nature of exacerbations, patients may find it difficult to identify the early stages of an exacerbation and to distinguish their increased COPD symptoms from daily variability or overlapping symptoms caused by flare-ups of comorbidities [[25], [26], [27], [28]]. Consequently, despite the use of an action plan, some patients may find it challenging to pinpoint the adequate time to initiate self-treatment of an exacerbation. Conversely, some ‘suboptimal adherent’ patients may deliberately have initiated self-treatment a few days earlier than instructed by the action plan. These patients may have recognised and acted toward early (and maybe very subtle) signs of an exacerbation, not detailed in their action plan (e.g. tightness, soreness of the chest) [29]. This implies the need for investigating a more personalised approach to COPD exacerbation self-management, such as further tailoring the action plan to the patient's symptom pattern during an exacerbation. Our data indicates the potential for introducing some flexibility into the action plans' utilisation and design, particularly within the limits of suboptimal adherence. For example, patients could be allowed a minor leeway (i.e. early start or slight delay) in initiating self-treatment, aligning with their individual needs, preferences and competences while still achieving meaningful health outcomes. Furthermore, for patients with limited health literacy, a simplified ‘one-step’ action plan can be considered, instructing patients to contact their healthcare professional for support when they experience poorly recognisable symptoms [30]. This presumably results in a slight delay in initiating treatment, but still within the predefined limits of suboptimal adherence to the action plan. Moreover, as anticipated, patients classified as ‘treatment outside the actual exacerbation period’ demonstrated low numbers of exacerbation-related health outcomes. Consequently, the decision to omit this adherence category from regression analyses concerning these health outcomes was deemed justified.

We are not able to explain the significantly better change from baseline dyspnoea CRQ domain score for patients classified as ‘treatment outside the actual exacerbation period’. In hindsight, we feel that the inclusion of periodically assessed health outcomes (e.g. CRQ and HADS) may be less relevant to explore, as these assessments were assessed at fixed time points and not directly linked to the COPD exacerbations. They could have been assessed several months after the end of an exacerbation. It is therefore less likely that patients' adherence to exacerbation action plans will have directly affected these health outcomes. Furthermore, there was no association found between adherence and the number of COPD exacerbations. This was expected, because the exacerbation action plan only instructs patients to start self-treatment in the event of a significant increase in respiratory symptoms and therefore does not affect the exacerbation frequency [11].

To improve patients' adherence and tackle significant non-adherence to COPD exacerbation action plans, there is a need to better understand the multifactorial underlying causes of non-adherence and how to tailor possible solutions accordingly [13]. Patients' adherence is known to be personally influenced by many disease-related, social, and psychological factors, such as the patients' health status, health literacy, health beliefs, and readiness to change [[31], [32], [33], [34], [35]]. Future research directed toward patterns of patients' adherence to exacerbation action plans at a more individual level over time could be really helpful to gain insight into how patients behave and act when their symptoms worsen. This can guide the implementation of personalised strategies that aim to elevate patients' adherence to exacerbation action plans, e.g. patient-tailored support from healthcare professionals, and training of self-management skills to elicit patients' adequate action plan use. Providing additional tailored support by e.g. case-managers may help patients to evaluate and tackle potential barriers of adherence, to learn from their previous exacerbation events, and to develop and master self-treatment decision making to act promptly in the course of exacerbations [30]. On top of that, in patients who are tech savvy, digital technology could potentially improve patients' adherence to action plans by facilitating the provision of tailored information, sending reminders and adapting the self-management intervention content to patients' needs, preferences and competencies [16,[36], [37], [38]]. Leveraging newer technologies in future self-management intervention studies, including digital innovations and the use of artificial intelligence (e.g. machine learning techniques), offers substantial promise in facilitating patients by predicting impeding exacerbations using individual patient symptom data [[39], [40], [41]]. This can help patients to recognise and start early treatment of exacerbations, thereby enhancing clinical decision making and patients’ adherence to exacerbation action plans.

Our study has several strengths and limitations. The main strength of this study was that we assessed multiple adherence categories rather than assessing adherence in a binary way. This yielded more detailed insights into patients' COPD exacerbation action plan usage. Furthermore, the combined population of two RCTs including patients with and without comorbid conditions, provided a relatively large sample size and reflects a heterogenous population of COPD patients, typical for usual care, which enhances the generalisability of our results. On the other hand, using multiple adherence categories led to a limited number of patients per adherence category. Another limitation of our study is the challenge posed by simultaneously assessing patients' adherence and exacerbations while evaluating these associations based on average observations during the one-year follow-up period. This approach fails to consider potential changes in adherence and (exacerbation-related) health outcomes over time. Our results therefore serve as a first step toward improved understanding of patients' adherence to COPD exacerbation action plans and associated health outcomes. Future studies in which both patterns of adherence to COPD exacerbation action plans and patterns of (exacerbation-related) health outcomes over time can be analysed and tested for associations, e.g., by using group-based trajectory modelling analyses [42], may provide a more in-depth understanding. Furthermore, our secondary analysis involved existing data from two RCTs, that were primarily designed to evaluate the effectiveness of self-management exacerbation action plans [11,12,19], and not patients’ adherence to these action plans. These considerations emphasize the need for a careful interpretation of our findings.

## 5 Conclusions

Our results demonstrate that patients' adherence to COPD exacerbation action plans is associated with fewer exacerbation days and a shorter duration per exacerbation compared to patients who initiated self-treatment with significant delay or not at all. Notably, suboptimal adherence still appears to be associated with comparable health benefits to optimal adherence. This highlights the potential for introducing some flexibility into the action plans' utilisation and design, the importance of patients' adherence in timely using exacerbation action plans, and the need for further patient-tailoring COPD self-management interventions. COPD self-management interventions should therefore include personalised strategies that aim to elevate patients' adherence to exacerbation action plans, and thereby improve patients’ health outcomes.

## CRediT authorship contribution statement

**Jade Schrijver:** Conceptualization, Methodology, Formal analysis, Writing - original draft, Writing - review & editing, Visualization, Project administration. **Tanja Effing:** Conceptualization, Methodology, Investigation, Writing - original draft, Writing - review & editing, Supervision, Funding acquisition. **Joanke van Helden:** Conceptualization, Methodology, Formal analysis, Writing - review & editing. **Job van der Palen:** Conceptualization, Methodology, Writing - review & editing, Supervision. **Paul van der Valk:** Conceptualization, Writing - review & editing. **Marjolein Brusse-Keizer:** Conceptualization, Writing - review & editing. **Anke Lenferink:** Conceptualization, Methodology, Investigation, Writing - original draft, Writing - review & editing, Supervision, Funding acquisition.

## Ethics statement

The COPE studies were approved by local ethics committees and registered (COPE II: NTR325; COPE-III: ACTRN12612000514808). All patients gave written informed consent before study participation.

## Data availability statement

Data that support the findings of this study are available upon request (corresponding author, J. Schrijver).

## Funding

This work was supported by Dutch Foundation for Asthma Prevention, Lung Foundation Netherlands (COPE-II study: grant number 3.4.02.12; COPE-III study: grant number 3.4.11.061), Lung Foundation Australia (Australian Lung Foundation Boehringer Ingelheim COPD Research Fellowship 2010), Repat Foundation, and GlaxoSmithKline (unrestricted grant).

## Declaration of competing interest

The authors declare that they have no known competing financial interests or personal relationships that could have appeared to influence the work reported in this paper.

## References

[1] Safiri S., Carson-Chahhoud K., Noori M. (2022). Burden of chronic obstructive pulmonary disease and its attributable risk factors in 204 countries and territories, 1990-2019: results from the Global Burden of Disease Study 2019. BMJ.

[2] Ramsey S.D., Sullivan S.D. (2003). The burden of illness and economic evaluation for COPD. Eur. Respir. J..

[3] Global Initiative for Chronic Obstructive Lung Disease (2023).

[4] Bourbeau J., van der Palen J. (2009). Promoting effective self-management programmes to improve COPD. Eur. Respir. J..

[5] Effing T.W., Bourbeau J., Vercoulen J. (2012). Self-management programmes for COPD: moving forward. Chron. Respir. Dis..

[6] Schrijver J., Lenferink A., Brusse-Keizer M. (2022). Self-management interventions for people with chronic obstructive pulmonary disease. Cochrane Database Syst. Rev..

[7] Lorig K.R., Holman H.R. (2003). Self-management education: history, definition, outcomes, and mechanisms. Ann. Behav. Med..

[8] Michie S., Richardson M., Johnston M. (2013). The behavior change technique taxonomy (v1) of 93 hierarchically clustered techniques: building an international consensus for the reporting of behavior change interventions. Ann. Behav. Med..

[9] Jalota L., Jain V.V. (2016). Action plans for COPD: strategies to manage exacerbations and improve outcomes. Int. J. Chron. Obstruct. Pulmon. Dis..

[10] Lenferink A., Brusse-Keizer M., van der Valk P. (2017). Self-management interventions including action plans for exacerbations versus usual care in patients with chronic obstructive pulmonary disease. Cochrane Database Syst. Rev..

[11] Lenferink A., van der Palen J., van der Valk P.D. (2019). Exacerbation action plans for patients with COPD and comorbidities: a randomised controlled trial. Eur. Respir. J..

[12] Effing T., Kerstjens H., van der Valk P. (2009). (Cost)-effectiveness of self-treatment of exacerbations on the severity of exacerbations in patients with COPD: the COPE II study. Thorax.

[13] van Boven J.F., Lavorini F., Dekhuijzen P.N. (2017). Urging Europe to put non-adherence to inhaled respiratory medication higher on the policy agenda: a report from the First European Congress on Adherence to Therapy. Eur. Respir. J..

[14] Choi J.Y., Chung H.I.C., Han G. (2014). Patient outcomes according to COPD action plan adherence. J. Clin. Nurs..

[15] Bischoff E.W., Hamd D.H., Sedeno M. (2011). Effects of written action plan adherence on COPD exacerbation recovery. Thorax.

[16] Farias R., Sedeno M., Beaucage D. (2019). Innovating the treatment of COPD exacerbations: a phone interactive telesystem to increase COPD Action Plan adherence. Br. Med. J. Open Respir. Res..

[17] Bucknall C.E., Miller G., Lloyd S.M. (2012). Glasgow supported self-management trial (GSuST) for patients with moderate to severe COPD: randomised controlled trial. Br. Med. J..

[18] Schrijver J., Effing T.W., Brusse-Keizer M. (2021). Predictors of patient adherence to COPD self-management exacerbation action plans. Patient. Educ. Couns..

[19] Lenferink A., Frith P., van der Valk P. (2013). A self-management approach using self-initiated action plans for symptoms with ongoing nurse support in patients with Chronic Obstructive Pulmonary Disease (COPD) and comorbidities: the COPE-III study protocol. Contemp. Clin. Trials.

[20] Rabe K.F., Hurd S., Anzueto A. (2007). Global strategy for the diagnosis, management, and prevention of chronic obstructive pulmonary disease: GOLD executive summary. Am. J. Respir. Crit. Care Med..

[21] Anthonisen N., Manfreda J., Warren C. (1987). Antibiotic therapy in exacerbations of chronic obstructive pulmonary disease. Ann. Intern. Med..

[22] Rodriguez-Roisin R. (2000). Toward a consensus definition for COPD exacerbations. Chest.

[23] Wilkinson T.M.A., Donaldson G.C., Hurst J.R. (2004). Early therapy improves outcomes of exacerbations of chronic obstructive pulmonary disease. Am. J. Respir. Crit. Care Med..

[24] Trappenburg J.C., Monninkhof E.M., Bourbeau J. (2011). Effect of an action plan with ongoing support by a case manager on exacerbation-related outcome in patients with COPD: a multicentre randomised controlled trial. Thorax.

[25] Effing T.W., Kerstjens H.A.M., Monninkhof E.M. (2009). Definitions of exacerbations: does it really matter in clinical trials on COPD?. Chest.

[26] Montes de Oca M., Laucho-Contreras M. (2018). Is it time to change the definition of acute exacerbation of chronic obstructive pulmonary disease? What do we need to add?. Medical Sciences.

[27] Jenkins C.R. (2021). Towards precision in defining copd exacerbations. Breathe.

[28] Sapey E., Bafadhel M., Bolton C.E. (2019). Building toolkits for COPD exacerbations: lessons from the past and present. Thorax.

[29] Williams V., Hardinge M., Ryan S. (2014). Patient’s experience of identifying and managing exacerbations in COPD: a qualitative study. NPJ Prim. Care Respir. Med..

[30] Effing T.W., Lenferink A., Moy M.L., Blackstock F., Nici L. (2020). Enhancing Patient Engagement in Pulmonary Healthcare: the Art and Science.

[31] Bourbeau J., Bartlett S. (2008). Patient adherence in COPD. Thorax.

[32] Krauskopf K., Federman A.D., Kale M.S. (2016).

[33] Kale M.S., Federman A.D., Krauskopf K. (2015). The association of health literacy with illness and medication beliefs among patients with chronic obstructive pulmonary disease. PLoS One.

[34] Blackstock F.C., Wallack R.Z., Nici L. (2016). Why don't our patients with chronic obstructive pulmonary disease listen to us? The enigma of nonadherence. Ann Am Thorac Soc.

[35] Russell S., Ogunbayo O.J., Newham J.J. (2018). Qualitative systematic review of barriers and facilitators to self-management of chronic obstructive pulmonary disease: views of patients and healthcare professionals. NPJ Prim. Care Respir. Med..

[36] Bourbeau J., Farias R. (2018). Making sense of telemedicine in the management of COPD. Eur. Respir. J..

[37] Fry J.P., Neff R.A. (2009). Periodic prompts and reminders in health promotion and health behavior interventions: systematic review. J. Med. Internet Res..

[38] Sloots J., Bakker M., van der Palen J. (2021). Adherence to an ehealth self-management intervention for patients with both COPD and heart failure: results of a pilot study. Int. J. Chron. Obstruct. Pulmon. Dis..

[39] Gonem S., Janssens W., Das N. (2020). Applications of artificial intelligence and machine learning in respiratory medicine. Thorax.

[40] De Ramón Fernández A., Ruiz Fernández D., Gilart Iglesias V. (2022). Analyzing the use of artificial intelligence for the management of chronic obstructive pulmonary disease (COPD). Int. J. Med. Inform..

[41] Watson A., Wilkinson T.M.A. (2022). Digital healthcare in COPD management: a narrative review on the advantages, pitfalls, and need for further research, Ther. Adv. Respir. Dis..

[42] Jennings W.G., Meade C. (2016).

[43] Guyatt G., Berman L., Townsend M. (1987). A measure of quality of life for clinical trials in chronic lung disease. Thorax.

[44] Zigmond A.S., Snaith R.P. (1983). The hospital anxiety and depression scale. Acta Psychiatr. Scand..

[45] Spinhoven P., Ormel J., Sloekers P.P.A. (1997). A validation study of the hospital anxiety and depression scale (HADS) in different groups of Dutch subjects. Psychol. Med..

[46] Munari A.B., Gulart A.A., Dos Santos K. (2018). Modified medical research Council dyspnea scale in GOLD classification better reflects physical activities of daily living. Respir. Care.

